# Progress in the Implementation of the secondary risk of suicide prevention program: a descriptive study at 12-month follow-up

**DOI:** 10.1192/j.eurpsy.2025.2369

**Published:** 2025-08-26

**Authors:** M. Valtueña Garcia, J. Sánchez-Adsuara, M. Mallo Caño, C. Moreno Blanco, C. Vera Varela, E. M. Sánchez Morla, I. Pérez Guerrero, D. Martínez Bermejo, J. Sánchez Hernández, J. Gómez Puebla, B. Arribas Domingo, M. Alonso García, M. Lorente Williams, M. Á. Gil Agero, M. L. Barrigón

**Affiliations:** 1 Psychiatry; 2 Mental health specialist nurse -Psychiatry; 3 Clinical psychology, Institute of Psychiatry and Mental Health-, Hospital General Universitario Gregorio Marañón.; 4 Faculty of CC. Health (Psychology) Rey Juan Carlos University; 5 Mental health research. General health psychologist and neuropsychologist., Institute of Psychiatry and Mental Health-, Hospital General Universitario Gregorio Marañón.; 6 Psychiatry, Gregorio Marañón Health Research Institute (IiSGM); 7 Resident internal psychologist - Clinical psychology; 8 Nurse -Psychiatry; 9 Resident intern physician - Psychiatry; 10 Social work, Institute of Psychiatry and Mental Health-, Hospital General Universitario Gregorio Marañón.; 11 IiSGM; 12 CIBERSAM; 13 Faculty of medicine. Complutense University of Madrid, Madrid, Spain

## Abstract

**Introduction:**

Suicide is one of the leading causes of preventable death.

The PRISURE program is developed with a series of objectives and actions aligned with the Mental Health Strategy of the National Health System - (Spain), Strategic Plan for Mental Health and Addictions of the Community of Madrid 2022-2024 and the Prevention Plan suicide in the community of Madrid 2022-2026, based on the experience of the suicide risk prevention program developed between 2014 and 2023 at the Retiro Mental Health Center (CSM) of the Institute of Psychiatry and Mental Health of the General University Hospital Gregorio Marañón.

**Objectives:**

Presentation of a secondary suicide prevention program in the Community of Madrid with 10 years of implementation and reinforcement of the therapeutic team in the last year.

Treatment outcomes, assessment of patients’ suicide risk progression during follow-up, referral to patient discharge, and outcome indicators in the past year are measured.

**Methods:**

Description of the functioning of the PRISURE program and descriptive study of sociodemographic and clinical characteristics, suicidal crises, evolution and discharge referrals, of all patients treated in PRISURE. The program’s performance indicators, as well as its results, are evaluated over one year from its implementation.

**Results:**

Sociodemographic and clinical characteristics are analyzed, including psychometric evaluation at baseline, 3, 6, 9, and 12 months after referral to PRISURE from August/2023 to August/2024. The suicide risk profile, treatment adherence, program implementation indicators and initial results are evaluated.

**Conclusions:**

PRISURE is a comprehensive care process that includes the prevention, intervention and postvention of suicidal behavior.

It includes interventions indicated for the prevention of suicidal behavior aimed at people in whom relevant signs or symptoms that anticipate the development of a mental disorder, or biological or psychological markers that indicate a high suicidal risk, have been identified.

PRISURE encompasses a set of activities aimed at early detection and indicated prevention, support and care of suicidal behavior, as well as research and promotion of mental health.

**Disclosure of Interest:**

None Declared

